# Supplementary material for: Extended-Spectrum β-Lactamase Genes Traverse the Escherichia coli Populations of Intensive Care Unit Patients, Staff, and Environment
Source: Microbiol Spectr. 2023 Mar 14;11(2):e05074-22. doi: 10.1128/spectrum.05074-22 (PMC10100714; doi:10.1128/spectrum.05074-22)
Supplement: Supplemental file 1 — Tables S1 to S10 and Fig. S1. Download spectrum.05074-22-s0001.pdf, PDF file, 0.5 MB [file spectrum.05074-22-s0001.pdf]

Extended-spectrum β-lactamase genes traverse the *Escherichia coli* populations of ICU patients, staff and environment

Supplementary Tables and Figure

Table S1: Environmental sampling locations.

Bed units	Communal areas
bed rail and regulator	computer: keyboard, mouse
ventilator (humidifier, display screen, connector)	phone
ECG monitor	barcode scanner
micropump	blood sample rack
switch button	medical record rack
nebuliser	sinks and pipe
stethoscope, flashlight	water dispenser
inner wall of hanging tower and drawer handle	cabinet and table
computer: keyboard, mouse	switch buttons
lockers	fibrobronchoscope
treatment vehicle	refrigerator
air disinfector	rescue vehicle
door	defibrillator
bed curtain	blood gas analyser
sink: tap	ultraviolet steriliser
sink: faucet surface	blood filter
sink: pool table	ECG machine
sink: drain	non-invasive ventilator
sink: water pipe	laryngoscope
	oxygen tank
	ice machine
	cleaning cart
	mop handle
	door handles

Table S2: Detection of DETEC-P793 plasmids by PCR.

Primer name	Sequence (5’-3’)	Product size (bp)	Plasmid detected
repDETEC13-FW	CCTGTTGCTTTGTTGAGGCT	592	pDETEC13
repDETEC13-RV	AGATTGGTGCGGGGTCTTTA		
RH1651	TTAGCACCCGAAGAGCAGAT	948	pDETEC14
RH1652	CTGACGCAACTCCCTGATG		
FIA-FW	CCATGCTGGTTCTAGAGAAGGTG	462	pDETEC15
FIA-RV	GTATATCCTTACTGGCTTCCGCAG		
repDETEC16-FW	CACGGATGATCTCGCTTTCG	760	pDETEC16
repDETEC16-RV	GCGCTTAGACTTAGAACCGC		

All PCRs were performed with an annealing temperature of 60°C.

Primers repDETEC13-FW/RV and repDETEC16-FW/RV were designed for this study.

Primers RH1651/1652 have been published in Moran *et al* 2015 (PMID 25819400) and FIA-FW/RV have been published in Carattoli *et al* 2005 (PMID 15935499).

Table S3: Overview of complete[1] genomes generated in this study.

Isolate[2]	Date	Source	Assembly[3]	ST	#ARGs[4]	#plasmids
DETEC-P61	01/08/2019	rectal swab	FUM[5]	48	22	6
DETEC-P1056	22/10/2019	rectal swab	FU	58	2	6
DETEC-S586	01/09/2019	rectal swab	U	69	5	10
DETEC-S589	01/09/2019	rectal swab	U	95	3	2
DETEC-E480	19/08/2019	switch button	FU	131	14	6
DETEC-P622	03/09/2019	rectal swab	FU	131	9	6
DETEC-S566	01/09/2019	rectal swab	FU	131	3	2
DETEC-P351	13/08/2019	rectal swab	U	167	7	6
DETEC-E223	06/08/2019	bed curtain	UM[6]	167	10	2
DETEC-S560	01/09/2019	rectal swab	U	167	7	5
DETEC-S792	17/09/2019	rectal swab	FUB	174	5	3
DETEC-S565	01/09/2019	rectal swab	U	196	6	5
DETEC-E601	03/09/2019	sink countertop	FU	345	30	4
DETEC-P169	06/08/2019	rectal swab	U	410	11	2
DETEC-P829	24/09/2019	rectal swab	FU	453	22	3
DETEC-P80	01/08/2019	rectal swab	U	744	23	4
DETEC-C31	15/10/2020	clinical sample	FU	1011	10	4
DETEC-E1070	22/10/2019	sink drain	U	1193	7	2
DETEC-P793	12/09/2019	rectal swab	U	1193	2	7
DETEC-P836	24/09/2019	rectal swab	U	1193	7	2
DETEC-E1005	15/10/2019	sink drain	U	1193	7	2
DETEC-P881	08/10/2019	oral swab	U	1236	14	2
DETEC-P649	05/09/2019	rectal swab	UM[7]	2003	9	8
DETEC-P666	10/09/2019	oral swab	FU	2003	2	12

[1] all complete genomes contained only circular chromosome and plasmid sequences

[2] DETEC-E = ICU environment, DETEC-P = ICU patient, DETEC-S = ICU staff, DETEC-C = clinical specimen

[3] assembly methods: U = unicycler, UM = unicycler with small plasmids finalised manually, FU = flye > unicycler, FUB = flye > unicycler bold mode, FUM = flye > unicycler with small plasmids finalised manually

[4] non-duplicate number of acquired antibiotic resistance genes

[5] pDETEC40 and pDETEC42 were resolved using contig coverage and comparison to closely related plasmids in the GenBank non-redundant nucleotide database.

[6] pDETEC12 was closed by joining two linear contigs. Comparison to pDETEC12 from strain DETEC-S560 and pCERC2 (GenBank accession KX291024) confirmed the assembly.

[7] pDETEC4 was closed by joining two linear contigs. Comparison to other pDETEC4 sequences in this dataset confirmed the assembly.

Table S4: Reference genomes used for phylogenetic analyses.

ST	# isolates	Alignment reference	Alignment type	Phylogeny available
1193	14	MCJCHV-1 (GCF_003344465.1)	Recombination removed	Yes
1236	5	DETEC-P881 hybrid assembly	Recombination removed	Yes
131	25	EC958 (GCF_000285655.3)	Recombination removed	Yes
167	6	51008369SK1 (GCF_003254065.1)	Recombination removed	Yes
2003	6	DETEC-P666 hybrid assembly	Recombination removed	Yes
345	12	DETEC-E601 hybrid assembly	Recombination removed	Yes
410	11	YD786 (GCF_001442495.1)	Recombination removed	Yes
453	5	DETEC-P829 hybrid assembly	Recombination removed	Yes
744	8	DETEC-P80 hybrid assembly	Recombination removed	Yes
48	4	DETEC-P61 hybrid assembly	Recombination removed	Yes
648	4	DETEC-P676 Illumina assembly	Recombination removed	Yes
69	4	DETEC-S586 hybrid assembly	Recombination removed	Yes
10	3	DETEC-S581 Illumina assembly	Full genome	No
13	2	DETEC-C13 Illumina assembly	Full genome	No
174	2	DETEC-S792 hybrid assembly	Full genome	No
196	2	DETEC-S565 hybrid assembly	Full genome	No
354	2	DETEC-P842 Illumina assembly	Full genome	No
38	3	DETEC-P152 Illumina assembly	Full genome	No
393	2	DETEC-C10 Illumina assembly	Full genome	No
4456	2	DETEC-C2 Illumina assembly	Full genome	No
457	3	DETEC-S592 Illumina assembly	Full genome	No
5416	2	DETEC-P817 Illumina assembly	Full genome	No
58	2	DETEC-P1056 hybrid assembly	Full genome	No

Table S5: CTX-M genes and IS*Ecp1* transposition unit characteristics in *E. coli* isolates.

Isolate	Genome	ST	CTX-M	TPU size	TSD	TPU location	Source of chromosomal TPU[2]
DETEC-C1	draft	1193	27	-	-	-	-
DETEC-C10	draft	393	27	-	-	-	-
DETEC-C11	draft	131	15	-	-	-	-
DETEC-C12	draft	1193	27	-	-	-	-
DETEC-C13	draft	13	55	-	-	plasmid; X1	-
DETEC-C14	draft	131	27	2927	TACAA	chromosome	-
DETEC-C15	draft	131	14, 15	3060	GCGGA	plasmid; Z	-
DETEC-C16	draft	12	14	4132	CATTA	plasmid; FII-2	-
DETEC-C17	draft	12742	55	3050	ATGAA	chromosome	H12 plasmid (AP023198)
DETEC-C18	draft	457	65	-	-	-	-
DETEC-C19	draft	1193	55	2971	TACTT	plasmid; I1	-
DETEC-C2	draft	4456	65	-	-	-	-
DETEC-C20	draft	58	14, 55	-	-	plasmid; FII-33	-
DETEC-C21	draft	131	27	2991	TTTTA	chromosome	-
DETEC-C22	draft	1193	3	2927	TTCTT	chromosome	-
DETEC-C23	draft	131	14	-	-	chromosome	-
DETEC-C23	draft	131	27	-	-	-	-
DETEC-C24	draft	131	27	2991	TTTTA	chromosome	-
DETEC-C25	draft	706	-	-	-	-	-
DETEC-C26	draft	2179	65	-	-	-	-
DETEC-C27	draft	131	27	2991	TTTTA	chromosome	-
DETEC-C28	draft	131	27	-	-	-	-
DETEC-C29	draft	131	27	-	-	-	-
DETEC-C3	draft	131	15	-	-	-	-
DETEC-C30	draft	410	15	-	-	-	-
DETEC-C31	complete	1011	55	2971	TACTT	plasmid; I1	-
DETEC-C32	draft	44	15	-	-	-	-
DETEC-C4	draft	131	14	-	-	-	-
DETEC-C5	draft	744	64	-	-	-	-
DETEC-C6	draft	453	-	-	-	-	-
DETEC-C7	draft	1193	55	2971	TACTT	plasmid; I1	-
DETEC-C8	draft	4456	55	2971	GTTTC	plasmid; FII-107:FIB-1	-
DETEC-C9	draft	13	55	-	-	plasmid; X1	-
DETEC-P526	draft	88	65	-	-	-	-
DETEC-E1005	complete	1193	55	2971	TATAT	chromosome	-
DETEC-E1007	draft	1193	55	2971	TATAT	chromosome	-
DETEC-E1010	draft	345	55	-	-	plasmid; FII-33	-
DETEC-E1024	draft	1193	55	2971	TATAT	chromosome	-
DETEC-E1029	draft	648	14	-	-	-	-
DETEC-E1033	draft	1236	55	2845	TGTTT	chromosome	-
DETEC-E1070	complete	1193	55	2971	TATAT	chromosome	-
DETEC-E1074	draft	69	65	-	-	-	-
DETEC-E159	draft	48	64	18201	TGTGT	chromosome	HI2 plasmid (MT773678)
DETEC-E161	draft	1196	55	-	-	-	-
DETEC-E223	complete	167	14	-	-	plasmid; FII-18	-
DETEC-E232	draft	744	55	-	-	plasmid; FII-33	-
DETEC-E249	draft	744	55	-	-	plasmid; FII-33	-
DETEC-E255	draft	744	55	-	-	plasmid; FII-33	-
DETEC-E257	draft	744	55	-	-	plasmid; FII-33	-
DETEC-E387	draft	744	55	-	-	plasmid; FII-33	-
DETEC-E455	draft	410	15	-	-	-	-
DETEC-E456	draft	410	15	-	-	-	-
DETEC-E457	draft	410	15	-	-	-	-
DETEC-E458	draft	410	15	-	-	-	-
DETEC-E459	draft	410	15	-	-	-	-
DETEC-E471	draft	167	-	-	-	-	-
DETEC-E480	complete	131	14, 55	-	-	plasmid; FII-33	-
DETEC-E481	draft	131	14, 55	-	-	plasmid; FII-33	-
DETEC-E521	draft	1485	55	2845	TGTTT	chromosome	-
DETEC-E600	draft	410	15	-	-	-	-
DETEC-E601	complete	345	55, 65	-	-	plasmid; FII-33	-
DETEC-E605	draft	345	55	-	-	plasmid; FII-33	-
DETEC-E628	draft	131	14	-	-	-	-
DETEC-E632	draft	131	14	-	-	-	-
DETEC-E674	draft	131	14	-	-	-	-
DETEC-E705	draft	345	55, 65	-	-	plasmid; FII-33	-
DETEC-E708	draft	345	65	-	-	-	-
DETEC-E710	draft	345	65	-	-	-	-
DETEC-E746	draft	2003	14	2855	TAGTA	chromosome	-
DETEC-E749	draft	2003	14	2855	TAGTA	chromosome	-

DETEC-E751	draft	2003	14	2855	TAGTA	chromosome	-
DETEC-E799	draft	167	14	-	-	-	-
DETEC-E810	draft	345	55, 65	-	-	plasmid; FII-33	-
DETEC-E812	draft	345	55, 65	-	-	plasmid; FII-33	-
DETEC-E814	draft	345	55, 65	-	-	plasmid; FII-33	-
DETEC-E816	draft	345	55, 65	-	-	plasmid; FII-33	-
DETEC-E835	draft	2003	14	2855	TAGTA	chromosome	-
DETEC-E846	draft	345	55, 65	-	-	plasmid; FII-33	-
DETEC-E869	draft	617	55	5800	TAATT	chromosome	I2 plasmid (LR890295)
DETEC-E880	draft	1193	55	2971	TATAT	chromosome	-
DETEC-E882	draft	1236	55	2845	TGTTT	chromosome	-
DETEC-E883	draft	1236	55	2845	TGTTT	chromosome	-
DETEC-P1008	draft	345	55, 65	-	-	plasmid; FII-33	-
DETEC-P1021	draft	648	14	-	-	-	-
DETEC-P1022	draft	5416	65	-	-	-	-
DETEC-P1030	draft	1236	55	2845	TGTTT	chromosome	-
DETEC-P1056	complete	58	14	3060	GCGGA	plasmid; Z	-
DETEC-P1067	draft	648	14	-	-	-	-
DETEC-P150	draft	162	14	-	-	-	-
DETEC-P152	draft	38	14	4477	TGAAA	chromosome	FII-2 plasmid (HM355591)
DETEC-P156	draft	4503	64	-	-	chromosome	-
DETEC-P169	complete	410	15	-	-	plasmid; F-type[x]	-
DETEC-P196	draft	156	55	2971	TCATA	plasmid; HI2	-
DETEC-P219	draft	354	14	3445	TAACC	chromosome	I-complex plasmid (CP054459)[4]
DETEC-P323	draft	410	15	-	-	-	-
DETEC-P351	complete	167	-	-	-	-	-
DETEC-P353	draft	167	-	-	-	-	-
DETEC-P363	draft	196	55	-	-	-	-
DETEC-P449	draft	5614	15	-	-	-	-
DETEC-P452	draft	410	15	-	-	-	-
DETEC-P475	draft	12546	65	-	-	-	-
DETEC-P477	draft	744	55	-	-	plasmid; FII-33	-
DETEC-P479	draft	453	55	-	-	plasmid; FII-33	-
DETEC-P530	draft	410	15	-	-	-	-
DETEC-P545	draft	131	14	-	-	-	-
DETEC-P546	draft	131	55	-	-	plasmid; FII-33	-
DETEC-P61	complete	48	64	18201	TGTGT	chromosome	HI2 plasmid (MT773678)
DETEC-P622	complete	131	14, 55	-	-	plasmid; FII-33	-
DETEC-P623	draft	131	14, 55	-	-	plasmid; FII-33	-
DETEC-P649	complete	2003	14	2855	TAGTA	chromosome	-
DETEC-P655	draft	69	65	-	-	-	-
DETEC-P656	draft	8492	65	-	-	-	-
DETEC-P666	complete	2003	14	2855	TAGTA	chromosome	-
DETEC-P671	draft	131	14	-	-	-	-
DETEC-P676	draft	648	14	-	-	-	-
DETEC-P72	draft	38	14	4477	TGAAA	chromosome	FII-2 plasmid (HM355591)
DETEC-P756	draft	3045	15	11384	TATCA	chromosome	H12 plasmid (AP023198)
DETEC-P793	complete	1193	55	2841	ATTCA	chromosome	-
			55	3350	ATGTT	plasmid; ColE2-like	-
DETEC-P794	draft	457	-	-	-	-	-
DETEC-P80	complete	744	55	-	-	plasmid; FII-33	-
DETEC-P817	draft	5416	65	-	-	-	-
DETEC-P829	complete	453	55	2971	TCATA	plasmid; HI2	-
DETEC-P836	complete	1193	55	2971	TATAT	chromosome	-
DETEC-P838	draft	3941	55	2845	TGTTT	chromosome	-
DETEC-P842	draft	354	55	-	-	-	-
DETEC-P849	draft	569	65	-	-	-	-
DETEC-P855	draft	450	15	2917	TTTTA	chromosome	-
DETEC-P876	draft	1193	55	2971	TATAT	chromosome	-
DETEC-P881	complete	1236	55	2845	TGTTT	chromosome	-
DETEC-S525	draft	131	14, 55	-	-	plasmid; FII-33	-
DETEC-S560	complete	167	55	2971	TACTT	plasmid; I1	-
DETEC-S561	draft	1193	3	2927	TTCTT	chromosome	-
DETEC-S562	draft	1722	15	-	-	chromosome	-
DETEC-S563	draft	453	55	-	-	-	-
DETEC-S564	draft	871	55	-	-	plasmid; X1	-
DETEC-S565	complete	196	55	2971	TACTT	plasmid; I1	-
DETEC-S566	complete	131	14	3060	GCGGA	plasmid; Z	-
DETEC-S567	draft	3052	15	11384	TAGCA	chromosome	H12 plasmid (AP023198)
DETEC-S568	draft	48	55	-	-	-	-
DETEC-S569	draft	117	55	-	-	-	-
DETEC-S570	draft	393	27	-	-	-	-

DETEC-S571	draft	69	14	3242	TATTA	chromosome	-
DETEC-S572	draft	202	15	-	-	-	-
DETEC-S573	draft	131	55	6397	TTGAT	chromosome	I1 plasmid (pDETEC69/pDETEC73)
DETEC-S575	draft	708	15	-	-	-	-
DETEC-S576	draft	5700	27	-	-	-	-
DETEC-S577	draft	453	55	-	-	-	-
DETEC-S578	draft	48	55	-	-	-	-
DETEC-S579	draft	2914	27	-	-	-	-
DETEC-S580	draft	38	14	-	-	chromosome	-
DETEC-S581	draft	10	14	-	-	-	-
DETEC-S582	draft	174	15	-	-	-	-
DETEC-S583	draft	624	14	-	-	-	-
DETEC-S584	draft	2690	55	-	-	-	-
DETEC-S585	draft	1249	14	-	-	-	-
DETEC-S586	complete	69	24	3271	AATCA	plasmid; X4	-
DETEC-S587	draft	1163	27	-	-	-	-
DETEC-S589	complete	95	14	3060	GCGGA	plasmid; Z	-
DETEC-S590	draft	10	55	-	-	plasmid; X1	-
DETEC-S591	draft	773	14	3502	TGATA	chromosome	-
DETEC-S592	draft	457	27	-	-	-	-
DETEC-S790	draft	1968	55	-	-	plasmid; FII-33	-
DETEC-S791	draft	93	55, 65	2971	TACTT	plasmid; I1	-
DETEC-S792	complete	174	55	-	-	plasmid; FII-2	-
DETEC-S897	draft	10	14	-	-	plasmid; Z	-

[1] - = not present or not determined.

[2] plasmid type is listed, along with a GenBank accession (in brackets) for a representative of the lineage that is the likely source of the chromosomal TPU.

[3] sub-type: FII-31:FII-36:FIA-4:FIB-58.

[4] the plasmid at accession CP054459 does not contain an IS*Ecp*1-*bla*$_{\text{CTX-M}}$ TPU, and represents an ancestral form of the plasmid that was the likely the source of this chromosomal TPU.

Table S6: Diversity amongst *E. coli* isolates of the same ST.

ST	Total isolates	Patient isolates	Environmental isolates	Staff isolates	Clinical isolates	Distinct people isolated from	Maximum SNPs between isolates of this [illegible]
131	25	5	5	3	12	6	597
1193	14	3	5	1	5	5	136
345	12	2	10	-	-	1	99
410	11	4	6	-	1	2	212
744	8	2	5	-	1	4	130
167	6	2	3	1	-	4	272
2003	6	2	4	-	-	2	8
1236	5	2	3	-	-	1	1
453	5	2		2	1	5	557
48	4	1	1	2	-	3	1504
648	4	3	1	-	-	2	178
69	4	1	1	2	-	4	311
10	3	-	-	3	-	3	20765
38	3	2	-	1	-	2	9016
457	3	1	-	1	1	3	7299
13	2	-	-	-	2	1	3
174	2	-	-	2	-	2	1
196	2	1	-	1	-	2	9949
354	2	2	-	-	-	2	9132
393	2	-	-	1	1	2	336
4456	2	-	-	-	2	1	1774
5416	2	2	-	-	-	2	1
58	2	1	-	-	1	2	8829

Table S7: Maximum cgSNPs amongst *E. coli* isolates of the same ST associated with a single patient

Patient ID	ST	# SNPs
P1	38	0
P5	48	0
P3	131	9
P14	131	3
P11	167	12
P8	345	99
P6	410	4
P23	648	12
P3	744	3
P23	1193	8
P27	1193	0
P24	1236	1
P17	2003	1

Table S8: GenBank plasmids containing the I1 plasmid TACTT-flanked IS*Ecp1*-*bla*$_{CTX-M-55}$ TPU

Plasmid	Accession	Host	Country	Year	Source	Size (bp)	*repA*[1]
p6607-69	CP045527	*S. sonnei*	Switzerland	2017	human faeces	89,848	pDETEC69
pST53-2	CP050747	*S. enterica* Typhimurium	China: Shanghai	2011	human faeces	88,006	pDETEC69
pHNRD174	KX246268	*E. coli*	China: Guangdong	≤2020	duck	86,207	pDETEC69
pXH990_3	CP019358	*E. coli*	China: Zhejiang	2016	human urine	86,028	pDETEC69
pD3-B	CP010142	*E. coli*	China: Jiangsu	2014	dog	90,074	pDETEC69
p628-CTXM	KP987217	*K. pneumoniae*	China: Beijing	2010	human cerebrospinal fluid	85,338	pDETEC69
pSKLX3330	KJ866866	*E. coli*	China: Zhejiang	≤2020	human urine	89,672	pDETEC69
pKP4823_3	KF790923	*K. pneumoniae*	China: Zhejiang	2019	human urine	86,182	pDETEC69
pURN1-2021	CP082826	*E. coli*	Kazakhstan	2021	human urine	86,164	pDETEC69
p14523A	CP074429	*Salmonella* sp.	China: Xinjiang	2016	human urine	85,862	pDETEC69
pRHBSTW-00218_2	CP056650	*E. hormachei*	United Kingdom	2017	wastewater influent	86,191	pDETEC69
pWP3-S18-ESBL-09	AP022037	*E. coli*	Japan	2018	wastewater effluent	88,528	pDETEC69
pKP4823_3	CP082793	*K. pneumoniae*	China: Zhejiang	2019	human urine	86,182	pDETEC69
pEC32-IncI1	CP085621	*E. coli*	China: Guangdong	2014	human urine	88,542	pDETEC69
pS29-IncI1	CP085700	*S. enterica*	China: Guangdong	2014	human faeces	88,502	pDETEC69
pKko009_2	CP091675	*E. coli*	Japan	2012	river water	89,404	pDETEC69
pEC24-3	CP060887	*E. coli*	China: Zhejiang	2017	human urine	86,975	pDETEC69
pEC20-3	CP060904	*E. coli*	China: Zhejiang	2017	human throat swab	86,975	pDETEC69
pEC19-3	CP060910	*E. coli*	China: Zhejiang	2017	human urine	86,975	pDETEC69
p2474-3	CP021208	*E. coli*	China: Hefei	2015	human blood	86,725	pDETEC73
pKFu019_1	CP091698	*E. coli*	Japan	2011	river water	86,740	pDETEC73
pEC7-3	CP060965	*E. coli*	China: Zhejiang	2016	human urine	89,379	pDETEC73
p2-3	CP091573	*S. enterica*	China: Zhejiang	2016	human faeces	86,718	pDETEC73
p20	CP099778	*S. sonnei*	Belgium	2015	human	86,718	pDETEC73
p72_2	CP101556	*K. pneumoniae*	China: Yunnan	2013	human urine	87,511	pDETEC73

[1] Indicates whether the plasmid contains the *repA* variant found in the pDETEC69 or pDETEC73 backbone.

Table S9: GenBank plasmids containing the Z plasmid GCGGA-flanked IS*Ecp1*-*bla*$_{CTX-M-14}$ TPU

Plasmid	Accession	Host	Country[1]	Year	Source	Size (bp)
pCT[2]	FN868832	*E. coli*	UK	2004	dairy farm calves	93,629
p2D-CTX-M-14	CP059004	*E. coli*	China	2019	human blood	96,449
pWP7-S17-ESBL-01_1	AP022174	*E. coli*	Japan	2017	wastewater plant effluent	94,776
pWP8-S17-ESBL-12_2	AP022224	*E. coli*	Japan	2017	wastewater plant effluent	87,171
pBEC1-S17-ESBL-07_1	AP022296	*E. coli*	Japan	2017	oceanic water	105,884
unnamed	LR890721	*K. pneumoniae*	Australia*	-	-	89,062
pJX1-2	CP064254	*K. pneumoniae*	China	2018	human	102,456
pF16EC0557-1	CP088388	*E. coli*	South Korea	2016	human blood	150,230
pF16EC0617-4	CP088378	*E. coli*	South Korea	2016	human blood	87,357
pF16EC0342-2	CP088412	*E. coli*	South Korea	2016	human blood	111,606
pF16EC0121-1	CP088449	*E. coli*	South Korea	2016	human blood	94,534
pE16EC0790-2	CP088518	*E. coli*	South Korea	2016	human blood	90,665
pC17EC0264-2	CP088619	*E. coli*	South Korea	2017	human blood	94,108
pD16EC1060-3	CP088610	*E. coli*	South Korea	2016	human blood	98,480
pB16EC1060-3	CP088732	*E. coli*	South Korea	2016	human blood	90,665
pB16EC0725-1	CP088777	*E. coli*	South Korea	2016	human blood	106,218
pB16EC0268-1	CP088807	*E. coli*	South Korea	2016	human blood	90,653
pA17EC0191-3	CP088824	*E. coli*	South Korea	2017	human blood	93,421
pA16EC0054-1	CP088870	*E. coli*	South Korea	2016	human blood	88,005
unnamed3	CP090250	*E. coli*	China	2018	human	90,981
p3	CP090538	*S. enterica*	China	2016	human	90,380
pEC-16-35-4	CP093229	*E. coli*	China	2016	human ascites	90,339
pEC9682-2	CP095273	*E. coli*	China	2021	human vaginal secretion	96,608
pPIB-2	CP090404	*Shigella* sp.	China	2014	human	87,994
P2	OX030730	*E. coli*	Spain	-	-	100,782
p69	CP099769	*S. sonnei*	Belgium	2018	human	111,597
pSH15sh99	KY471628	*S. sonnei*	China	2015	drinking water	104,285
pSH15sh104	KY471629	*S. sonnei*	China	2015	human faeces	104,285
RCS56_p	LT985270	*E. coli*	France*	-	-	90,206
RCS68_p	LT985278	*E. coli*	France*	-	-	86,943
pSH262-2	MG299128	*S. sonnei*	China	2016	human	109,845
pSH271-2	MG299131	*S. sonnei*	China	2016	water	109,845
pSH272-2	MG299133	*S. sonnei*	China	2016	human	109,845
pSH284-2	MG299147	*S. sonnei*	China	2016	human	109,845
pSH287-2	MG299151	*S. sonnei*	China	2016	human	109,845
p50579417_2	CP033883	*E. coli*	Norway	2012	human urine	88,005
unnamed	LR595871	*E. coli*	UK*	-	human faeces	94,061
unnamed	LR595872	*E. coli*	UK*	-	human faeces	96,306
unnamed	LR595877	*E. coli*	UK*	-	human faeces	111,594
unnamed	LR595880	*E. coli*	UK*	-	human faeces	96,305
unnamed	LR595888	*E. coli*	UK*	-	human faeces	96,306
unnamed	LR595889	*E. coli*	UK*	-	human faeces	94,296
pKP16-19-tet(A)	MN480462	*K. pneumoniae*	China	2016	human sputum	106,623
pSCU-103-3	CP054460	*E. coli*	USA	2015	human rectal swab	34,914

[1] * indicates where the submitting authors' location is listed, as a country of isolation was not stated in the GenBank entry.

[2] pCT is has a K-type replicon, indicating that recombination has likely resulted in the movement of the GCGGA-flanked IS*Ecp1*-*bla*$_{CTX-M-14}$ TPU and adjacent backbone sequence between plasmids with Z and K-type replicons.

Table S10: Complete plasmid sequences generated in this study.

**Type** Sub-type	Plasmid	Size (bp)	#ARGs	GenBank Accession
**F-type**				
FII-2	pDETEC44	82,435	5	CP116069
FII-16	pDETEC24	76,416	3	CP116102
FII-29	pDETEC80	81,583	2	CP116087
FII-33	pDETEC21	103,125	8	CP116113
FII-33	pDETEC61	93,520	8	CP116177
FII-33:N	pDETEC56	103,383	8	CP116183
FII-33:N	pDETEC60	82,763	3	CP116147
FII-35	pDETEC48	70,464	-	CP116077
FII-49	pDETEC57	13,744	1	CP116148
FIB-54:phage-plasmid	pDETEC37	142,064	9	CP116153
FII-1:FIA-1	pDETEC32	98,478	1	CP116124
FII-36:FIA-4	pDETEC5	116,501	5	CP116160
FII-18:FIB-1	pDETEC11[2]	154,454	8	CP116189
FII-18:FIB-1	pDETEC19[2]	159,599	7	CP116111
FII-18:FIB-1	pDETEC68	102,896	4	CP116095
FII-18:FIB-1	pDETEC77[1]	132,207	2	CP116072
FII-18:FIB-1	pDETEC88[2]	111,058	2	CP116199
FII-18:FIB-35	pDETEC81	90,243	-	CP116170
FII-107:FIB-1	pDETEC46	141,890	4	CP116075
FIA-1:FIB-10	pDETEC3	108,072	6	CP116104
FIA-1:FIB-10	pDETEC15	82,761	-	CP116118
FIA-6:FIB-20	pDETEC55	107,932	5	CP116146
FII-2:FIA-1:FIB-1	pDETEC38	128,466	3	CP116154
FII-18:FIA-5:FIB-1	pDETEC23[1]	178,682	6	CP116101
FII-18:FIA-5:FIB-1	pDETEC72[1]	187,674	5	CP116089
FII-18:FIA-2:FIB-8	pDETEC66[1]	194,772	-	CP116108
FII-novel:FIA-1:FIB-23	pDETEC25	151,807	8	CP116137
FII-31:FII-36:FIA-4:FIB-58	pDETEC2	113,066	6	CP116168
**I-complex**				
I1	pDETEC6	77,960	1	CP116161
I1	pDETEC14	87,921	-	CP116117
I1	pDETEC20	118,716	4	CP116112
Z	pDETEC33	86,916	-	CP116125
Z	pDETEC43	98,708	-	CP116068
Z	pDETEC45	81,965	-	CP116070
I1	pDETEC47	87,625	-	CP116076
I1	pDETEC67	118,588	5	CP116109
I1	pDETEC69	86,167	1	CP116096
I1	pDETEC73	90,981	1	CP116090
Z	pDETEC78	89,022	1	CP116073
Z	pDETEC79	87,662	1	CP116086
Z	pDETEC82	88,352	1	CP116171
I1	pDETEC89	86,715	1	CP116200
I1	pDETEC91	85,862	1	
**X-type**				
	pDETEC35	38,312	-	CP116127
	pDETEC36	30,896	-	CP116128
X1	pDETEC39	44,960	7	CP116155
X4	pDETEC49	42,640	1	CP116078
	pDETEC62	36,682	-	CP116178
**H-type**				
	pDETEC13	209,927	-	CP116116
HI2	pDETEC65	266,247	17	CP116107

**N-type**				
N2	pDETEC50	41,594	-	CP116079
**phage-plasmid**				
	pDETEC1	119,948	5	CP116167
	pDETEC34	47,568	-	CP116126
	pDETEC74	89,020	-	CP116091
	pDETEC87	113,249	-	CP116198
**θ-RNA**				
	pDETEC9	3,684	-	CP116164
	pDETEC10	3,174	-	CP116165
	pDETEC12	6,200	2	CP116097
	pDETEC17	5,631	-	CP116120
	pDETEC26	5,775	-	CP116129
	pDETEC28	4,593	-	CP116131
	pDETEC40	5,903	-	CP116156
	pDETEC42	3,263	-	CP116158
	pDETEC51	7,939	-	CP116080
	pDETEC53	4,715	-	CP116083
	pDETEC63	4,045	-	CP116179
	pDETEC64	3,731	-	CP116180
	pDETEC71	2,678	-	CP116099
	pDETEC75	6,647	-	CP116092
	pDETEC83	7,559	-	CP116172
	pDETEC90	3,537	-	CP116201
**θ-Rep**				
	pDETEC7	6,074	-	CP116162
	pDETEC8	4,073	-	CP116163
	pDETEC16	8,520	1	CP116119
	pDETEC18	4,060	-	CP116121
	pDETEC22	4,692	-	CP116114
	pDETEC27	5,167	-	CP116139
	pDETEC30	4,069	-	CP116133
	pDETEC41	4,867	-	CP116157
	pDETEC52	5,163	-	CP116082
	pDETEC54	4,059	-	CP116084
	pDETEC58	4,087	-	CP116150
	pDETEC84	5,728	-	CP116173
	pDETEC85	4,296	-	CP116174
**Rolling-circle**				
	pDETEC4	2,101	-	CP116105
	pDETEC29	4,396	-	CP116132
	pDETEC31	1,551	-	CP116135
	pDETEC59	1,552	-	CP116151
	pDETEC76	1,565	-	CP116093
**Untyped**				
	pDETEC70	4,036	-	CP116098
	pDETEC86	1,985	-	CP116175

[1] ColV plasmid

[2] ColBM plasmid

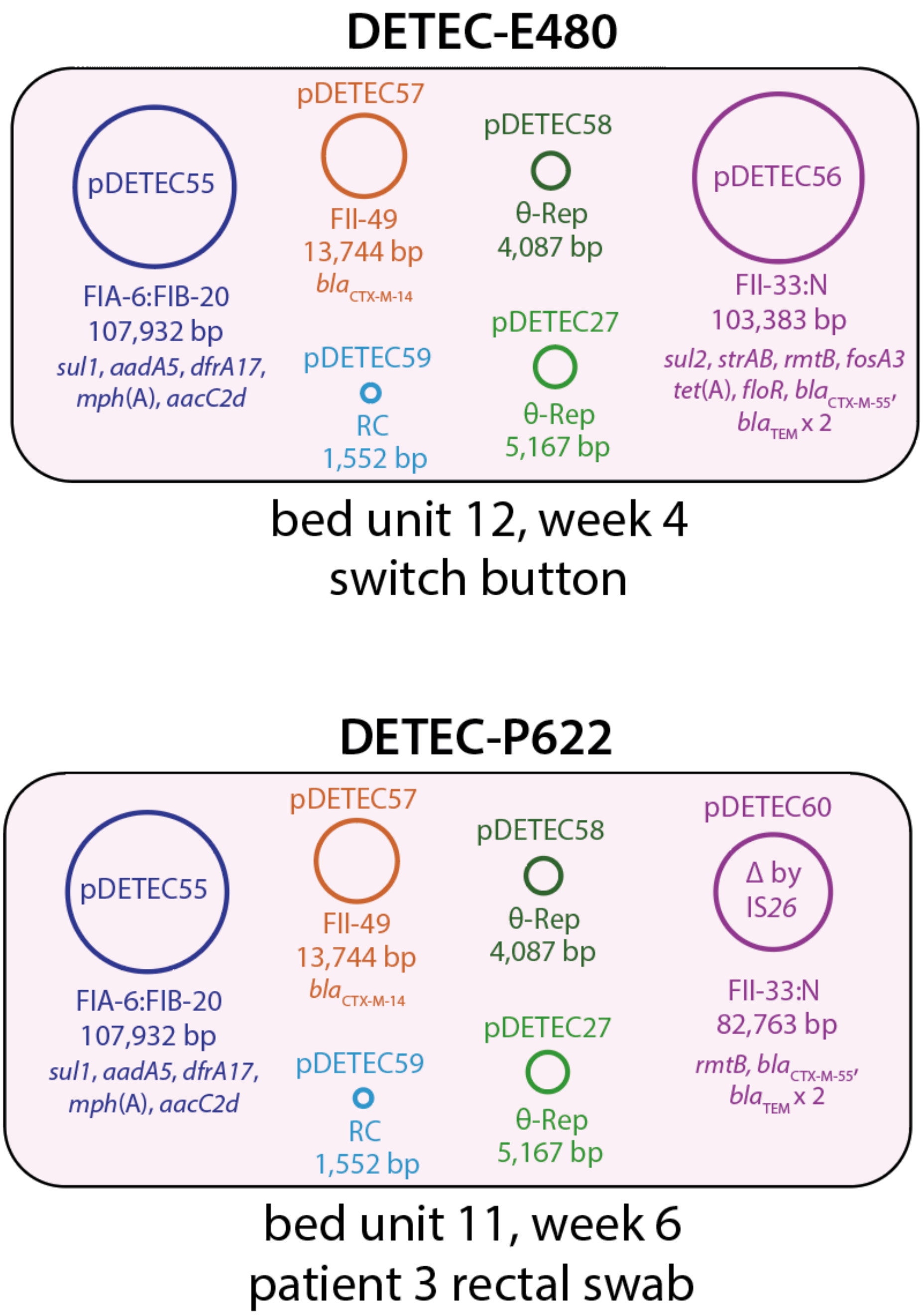

Figure S1: Plasmid content of ST131 isolates DETEC-E480 and DETEC-P622.
